# Discovery of a natural cyan blue: A unique food-sourced anthocyanin could replace synthetic brilliant blue

**DOI:** 10.1126/sciadv.abe7871

**Published:** 2021-04-07

**Authors:** Pamela R. Denish, Julie-Anne Fenger, Randall Powers, Gregory T. Sigurdson, Luca Grisanti, Kathryn G. Guggenheim, Sara Laporte, Julia Li, Tadao Kondo, Alessandra Magistrato, Mícheál P. Moloney, Mary Riley, Mariami Rusishvili, Neda Ahmadiani, Stefano Baroni, Olivier Dangles, Monica Giusti, Thomas M. Collins, John Didzbalis, Kumi Yoshida, Justin B. Siegel, Rebecca J. Robbins

**Affiliations:** 1 Biophysics Graduate Group, University of California, Davis, Davis, CA, USA.; 2 Genome Center, University of California, Davis, Davis, CA 95616, USA.; 3 Avignon University, INRAE, Avignon, France.; 4 Mars Wrigley, Hackettstown, NJ 07840, USA.; 5 Department of Food Science and Technology, The Ohio State University, Columbus, OH 43210, USA.; 6 Scuola Internazionale Superiore di Studi Avanzati, Trieste, Italy.; 7 Division of Theoretical Physics, Institut Ruđer Bošković, Zagreb, Croatia.; 8 Chemistry Department, University of California, Davis, Davis, CA 95616, USA.; 9 Graduate School of Informatics, Nagoya University, Chikusa, Nagoya, Japan.; 10 Consiglio Nazionale delle Ricerche, Istituto Officina dei Materiali, Scuola Internazionale Superiore di Studi Avanzati, Trieste, Italy.; 11 Microbiology Graduate Group, University of California, Davis, Davis, CA 95616, USA.; 12 Pritzker School of Molecular Engineering, The University of Chicago, Chicago, IL, USA.; 13 Centre d’Innovació, Recerca I Transferència en Tecnologia dels Aliments, CERTA-UAB Tecnio Grup, XIA-UAB, Animal and Food Science Department, Universidad Autònoma de Barcelona, Bellaterra, Spain.; 14 Mars Advanced Research Institute, Mars, Incorporated, Hackettstown, NJ 07840, USA.; 15 Department of Biochemistry and Molecular Medicine, University of California, Davis, Sacramento, CA 95616, USA.; 16 Mars Wrigley Global Innovation Center, Chicago, IL 60642, USA.

## Abstract

The color of food is critical to the food and beverage industries, as it influences many properties beyond eye-pleasing visuals including flavor, safety, and nutritional value. Blue is one of the rarest colors in nature’s food palette—especially a cyan blue—giving scientists few sources for natural blue food colorants. Finding a natural cyan blue dye equivalent to FD&C Blue No. 1 remains an industry-wide challenge and the subject of several research programs worldwide. Computational simulations and large-array spectroscopic techniques were used to determine the 3D chemical structure, color expression, and stability of this previously uncharacterized cyan blue anthocyanin-based colorant. Synthetic biology and computational protein design tools were leveraged to develop an enzymatic transformation of red cabbage anthocyanins into the desired anthocyanin. More broadly, this research demonstrates the power of a multidisciplinary strategy to solve a long-standing challenge in the food industry.

## Teaser

A 3D molecular arrangement and coordination of a minor anthocyanin from red cabbage creates a brilliant blue color.

## INTRODUCTION

**Fig. 1:**
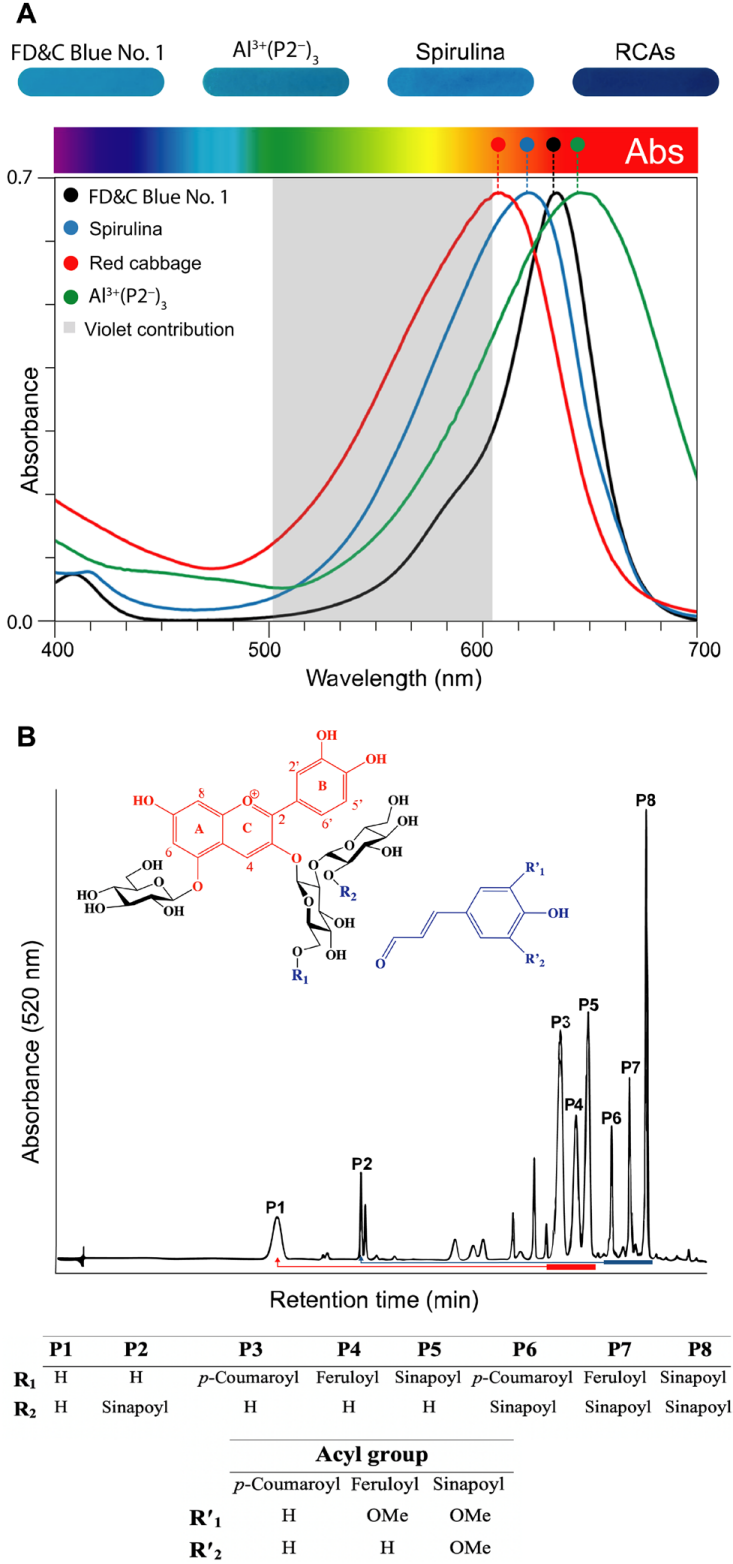
Color expression of four blue pigments and the structure of RCAs. (**A**) The UV-visible spectra depicted highlights λ_max_ and its respective location on the electromagnetic (EM) absorbance spectra. λ_max_ for FD&C Blue No. 1 is 630 nm, spirulina is 617 nm, RCAs at pH 8 are 608 nm, and the Al^3+^(P2^−^)_3_ complex at pH 7 is 640 nm. The violet contribution (VC) of each blue, defined as the absorption in the 500- to 600-nm range (depicted in gray), is shown. Values and calculations for VC, i.e., area under the curve (AUC), are provided in sections S1 and S2. (**B**) The red flavylium cation of the main anthocyanins in red cabbage is depicted as P1 to P8 on the HPLC-diode-array-detection (DAD) trace (520-nm detection) (method S11.2). RCAs display a structural homology (red and blue arrows) where the common building blocks are a cyanidin chromophore (highlighted in red), sugars, and small acyl groups (highlighted in blue). The sophorose moiety is bound at position 3, and a glucose is bound at position 5. The key differentiation is acyl groups (*p*-coumaroyl, feruloyl, and sinapoyl) and their substitution patterns on sugar 1 (Glc-1) or sugar 2 (Glc-2) of the sophorose.

Despite a long history of exploration, blue remains one of the most challenging colorants to obtain from any source and even more so from natural, edible sources (*1*–*7*). Blue is critical as it is necessary to produce other colors across the palette. Furthermore, the subtleties of color differences in different blues are of great importance in the area of blending colors (*8*). The two main artificial blue food colorants are brilliant blue (FD&C Blue No. 1) and indigotine (FD&C Blue No. 2), providing cyan (λ_max_ = 630 nm) and indigo (λ_max_ = 608 nm) hues, respectively. Naturally occurring food-based blues are limited and can be sourced from anthocyanins and a limited set of other blue colorants including phycocyanins (from *Spirulina* spp.) (λ_max_ = 615 to 620 nm) (*9*) and iridoid derivatives from huito (or gardenia) (λ_max_ = 590 to 610 nm) (*10*, *11*). However, to date, all known natural colorants have either a λ_max_ less than 630 nm, a large violet color contribution (absorbance in the range of 500 to 600 nm), or both (Fig. 1A, method S1.1, and figs. S1.1 and S1.2). Violet contributions affect the final color in blended colorants, e.g., blending natural blues with yellow generally results in a muddy green (fig. S2.1 and discussion S2.1) (*8*). Although green is abundant in nature, the chlorophyll chromophore is not stable or water soluble (section S2.1) and, therefore, has limited application (*12*). Achieving a cyan blue from natural sources that could be used as replacement for FD&C Blue No. 1 enabling a broader color palette has been a long-standing challenge to the food industry.

Anthocyanins display a large versatility in color expression because of a complex chemical equilibrium of colored and colorless forms (fig. S3.1 and discussion S3.1) (*13*–*17*). These naturally occurring chromophores are generally intensely red-colored under acidic conditions based on the flavylium cation (Fig. 1B) and change toward violet and blue as the pH increases. Concurrently, the colored forms are susceptible to fading by a combination of water addition (reversible) and autoxidation (irreversible), limiting their stability over time (*18*). A challenge with anthocyanins is the plethora of different molecules found in a single crop source, such as red cabbage (Fig. 1B) or purple sweet potato (*19*, *20*). Both well-established commodity crops generate blue colors at pH 7 to 9. However, it is a composite color because of the numerous individual anthocyanin molecules present and the various equilibrium forms at a given pH for each individual anthocyanin molecule (*10*, *19*, *20*). While the combined red cabbage anthocyanins (RCAs) will produce a vibrant and attractive blue color in pH-neutral solution, there is still a relatively large violet color contribution, limiting their utility as a natural FD&C Blue No. 1 replacement (Fig. 1A).

Previous efforts to identify the chemical structures and spectral properties of RCAs uncovered an interesting structural homology where the common building blocks are a cyanidin chromophore, a sophorose moiety bound at position 3, and a glucose bound at position 5 (Fig. 1B) (*21*–*25*). The key differentiation factors are the presence of hydroxycinnamic acids and their substitution patterns on sugar 1 (Glc-1) or sugar 2 (Glc-2) of the sophorose (Fig. 1B, table inset). Remarkably, one of the minor mono-acylated anthocyanins, Peak 2 (P2) was found to have a particularly high λ_max_ of 640 nm at pH 7 (*20*). While only a minor component within the RCA mixture, this unique compound warranted further investigation to understand the structural factors conferring its desirable spectral properties.

## RESULTS AND DISCUSSION

### Structural analysis of P2

Previous efforts relied on mass and retention time or established standards to make predictions of the P2 structure (*20*). Before conducting an in-depth investigation of the structural factors, which impart the unique spectral properties to P2, it was prudent to obtain a complete structural assignment of the compound. The structural assignment for P2 was determined by high-resolution electrospray ionization–time-of-flight mass spectrometry (ESI-TOF-MS) and a combination of ^1^H and ^13^C nuclear magnetic resonance (NMR), one-dimensional total correlation spectroscopy (1D TOCSY), correlation spectroscopy (COSY), nuclear Overhauser effect spectroscopy (NOESY), heteronuclear single-quantum correlation spectroscopy (HSQC), and heteronuclear multiple bond correlation (HMBC) experiments and proven to be 3-*O*-(2-*O*-(2-*O*-(*E*)-sinapoyl-β-d-glucopyranosyl)-β-d-glucopyranosyl)-5-*O*-β-d-glucopyranosylcyanidin (discussion S5.1, table S5.1, and figs. S5.1 to S5.9) (*26*, *27*). The results were consistent with the previously predicted structure, supporting that P2 is a regio-isomer of P5 where only the sinapoyl residue resides on a different sugar (Fig. 1B).

It is well established that cyanidin-based chromophores, such as P2, require a combination of neutral pH and complexation to a metal ion, such as an Al^3+^, at the ortho-dihydroxyphenyl moiety (Fig. 1B, positions 3′ and 4′) of the cyanidin chromophore to produce a blue color (*15*, *17*, *27*, *28*). To evaluate the effect of metal ion complexation with specific RCA components, P2, P5, and P8 were incubated with Al^3+^. The only difference between P2, P5, and P8 is the number and placement of sinapoyl moieties on the sophorose, enabling a direct comparison of the primary structural feature differentiating RCAs and their effect on color formation.

**Fig. 2:**
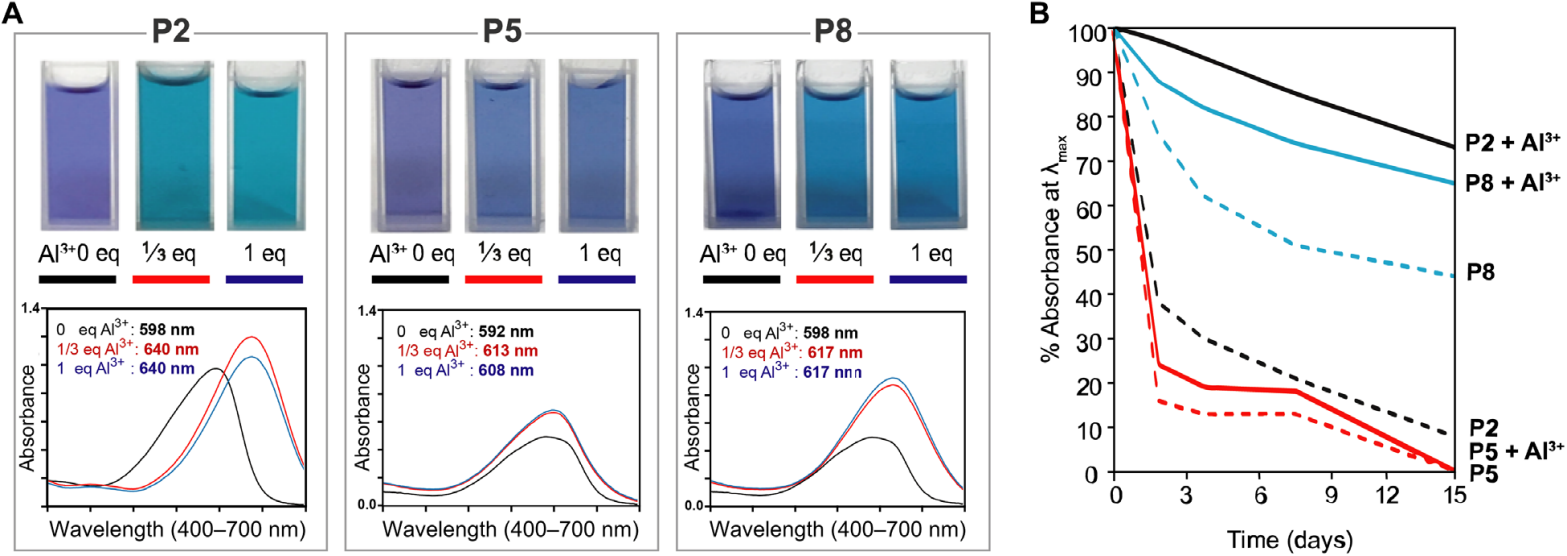
Absorption spectrum and stability sinapoyl containing RCAs. (**A**) Cuvettes with the corresponding visible spectrum, 400 to 700 nm, for P2, P5, and P8 at pH 7 with and without Al^3+^. The different equivalences of Al are color-coded: black = 0 eq, red = ^1^/_3_ eq, and blue = 1 eq. The λ_max_ values for 0 eq are 598 nm for P2, 596 nm for P5, and 598 nm for P8. Adding ^1^/_3_ eq Al^3+^ generates a λ_max_ value of 640 nm for P2, 602 nm for P5, and 617 nm for P8. Adding 1 eq of Al^3+^ does not appear to affect λ_max_ for P2, P5, or P8. (**B**) Stability (absorbance at λ_max_ of aqueous solutions monitored over time) of anthocyanins P2, P5, and P8 at pH 7 over time with (solid line) and without Al^3+^ (dashed line). The addition of Al^3+^ enhances the stability of both P2 and P8; however, it has a minor effect on the stability of P5. P8 has an inherent additional stability because of diacylation but is still surpassed by the stability of P2 + Al^3+^. Once the Al^3+^(P2^−^)_3_ complex is formed, it demonstrates a marked increase in stability that is thought to stem from the intermolecular interlocking of the P2 moieties (figs. S8.2 to S8.5).

The discrimination between the spectral properties of P2, P5, and P8 in the presence of Al^3+^ is highly unexpected (Fig. 2A). With only one-third equivalent of Al^3+^, the P2 solution gave the desired blue colored complex with a large bathochromic shift of >40 nm (λ_max_ at 640 nm; Figs. 1A and 2A, figs. S6.1 to S6.4, and discussion S6.2). In contrast, P8 and P5 had only modest shifts of <20 nm. This provides direct evidence that the sinapoyl moiety and its position (on Glc-2 in P2), despite its intrinsic inability to bind metal ions, play a critical role in the structure of the metal complex, leading to a very strong bathochromic shift.

**Fig. 3:**
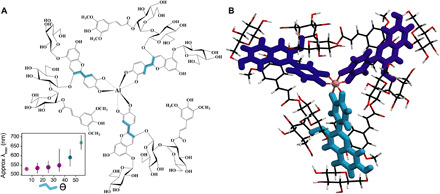
Proposed trimeric structure. (**A**) A proposed trimeric structure is shown in (B), where three P2 molecules form a propeller-like structure around the aluminum ion. NMR, MS, and Cotton effects of Al^3+^(P2^−^)_3_ were confirmed (details provided in sections S7, S8, and S10). Computational modeling indicates that the large bathochromic shift observed stems mainly from the enhanced distortion of angle θ, highlighted in blue on the molecular structure. Inset shows the calculated effect of λ_max_ as a function of θ angle distortion. The color of each circle is the average expected color, the size of the marker corresponds to average oscillator strengths, and error bars are computed as SD of λ_max_. The data for the figure correspond to cyanidin-3-glucoside in fully solvated water. Molecular structures were pruned by replacing the sugar with a methyl group and absorption spectra calculated with time-dependent density functional theory [6-311G(d,p) at B3LYP] (DFT-B3LYP) and fully detailed in section S10. (**B**) 3D representation and coordination of the anthocyanin P2 around the aluminum.

To elucidate this uniquely colored self-assembled structure of the three P2 molecules coordinated to aluminum metal (*29*), the circular dichroism (CD) spectrum of P2 and Al^3+^ was recorded in buffered solutions. With the addition of Al^3+^, P2 showed an exciton-type positive Cotton effect around λ_max_, while no such Cotton effect was observed without Al^3+^. These results indicate that Al^3+^ is critical to the coordination and building of the tertiary structure that exhibits a chiral arrangement of three P2 monomers around the Al^3+^ ion (Fig. 3, A and B, and section S8). By contrast, P5 displayed very minor Cotton effects (section S7.1, figs. S7.1 to S7.3, and table S7.1). Furthermore, negative-mode high-resolution ESI-TOF-MS analysis of the P2/Al solution at pH 7 provided evidence for molecular ion peaks at mass/charge ratio (*m/z*) = 1478.8629 and at *m/z* = 985.5760, which can be respectively attributed to [3×P2+Al−2H]^2−^ and [3×P2+Al–3H]^3−^, indicating the formation of a Al^3+^-P2 complex of 1:3 stoichiometry (Fig. 3A, section S7.2, and figs. S7.4 to S7.6). P5 did not give such multivalent molecular ion peaks by addition of Al^3+^ (fig. S7.5 to S7.7); however, P8 showed the similar divalent and trivalent ion peaks of [3×P8+Al–2H]^2−^ and [3×P8+Al–3H]^3−^ (figs. S7.8 to S7.10).

^1^H NMR measurement of the trimeric complex of Al^3+^(P2^−^)_3_ in D_2_O gave a very broad spectrum. However, numbers of signals in the lower field were relatively simple, indicating that the arrangement of the three P2 ligands within the Al complex might be highly symmetrical. Several signals were assignable, but analysis of NOE correlations was difficult (discussion S7.3, figs. S7.11 to S7.15, and table S7.2). Therefore, a structural analysis of Al^3+^(P2^−^)_3_ was undertaken by computational modeling. After extensive classical and first-principles molecular dynamics (MD) (*9*) simulations of P2, plausible geometries were constructed for threefold symmetric propeller-like structures of three P2 molecules around a single aluminum ion and minimized with first-principles calculations (discussion S8.1, method S8.1, fig. S8.1, and table S8.1). In all proposed configurations, the structure of the P2 cyanidin chromophore is distorted by a torsional displacement of the bond between the B and C rings (Fig. 3A, θ angle, and fig. S8.2, inset). A very interesting and remarkable result from the MD simulations, coupled with first-principles evaluation of excited states, is that torsional displacement of this bond causes the greatest change in the simulated color expression (*30*, *31*). The π-π electronic interaction between acyl and cyanidin motifs is not the main cause of the large color shift, as typically suggested. In this case, the HOMO and LUMO energies are more impacted by distortion of the cyanidin nucleus and the interactions among the ones coordinated to the same metal (excitonic effects) than by acyl-cyanidin interactions (discussions S8.1 and S8.2). However, the π-π interaction between the hydrophobic aromatic rings of the acyl and cyanin groups has a critical influence on the conformations: While intramolecular π-π stacking dominates in isolated P2 and P5 (discussion S8.3), the proposed Al^3+^(P2^−^)_3_ complex manifests intermolecular π-π stacking between adjacent P2 moieties (interlocking between P2 units shown in figs. S8.2 to S8.4), which is consistent with the strong Cotton effect observed by CD and the enhanced stability of the complex discussed below.

Among all RCAs, P2 uniquely displays two remarkable long-range NOE correlations between the cyanidin and sinapoyl residue in a solvent that does not favor π-stacking interactions (MeOH), indicating a folding of the molecule (figs. S5.3 and S5.4) (*21*). The disaccharide in the β-d-Glc(2-sinapoyl)-1,2-β-d-Glc-3-cyanidin sequence within monoacylated P2 likely provides optimal intramolecular spacing for enhanced conformational stability. Protection against hydration (water addition at position C2) was modest with monoacylated P5 (p*K*′_h_ = 2.7, versus 2.1 for nonacylated P1) but more pronounced with diacylated P8 (p*K*′_h_ = 3.7) (*15*, *21*, *28*). P2 deviates from this trend and is exceptionally resistant to water addition (p*K*′_h_ = 4.4) despite its monoacylated status (section S9.1, fig. S9.1, and table S9.1) (*14*, *15*). This resistance to hydration is reflected by the superior long-term stability of P2 compared to P5 and P8 and Al^3+^(P2^−^)_3_ compared to the P5 and P8 mixtures (Fig. 2B and fig. S6.3). In addition, although MD simulations of P2 and P5 show that, in both cases, folded conformations are favored, P2 displays a higher percentage of closed conformations than P5 (method S10.1, fig. S10.1, and discussion S10.1). It is hypothesized that the “tightly closed” P2 structure enables a closer approach to Al^3+^ (fig. S10.1c) and a trimeric arrangement around the metal ion. This arrangement causes an increase in the torsional distortion (angle θ) of the B-ring to C-ring bond, which enhances the bathochromic shift of the visible absorption band (Fig. 3A, inset). Although P8 is closely related to P2, its additional sinapoyl residue might provide steric hindrance limiting the simultaneous interaction of three P8 molecules with Al^3+^. As for P5, its more open structure (cyanidin-sinapoyl interactions weaker than in P2) might prevent the arrangement of anthocyanin molecules around Al^3+^, precluding formation of the Al^3+^(P5^−^)_3_ complex.

### Enzymatic enrichment of P2

**Fig. 4:**
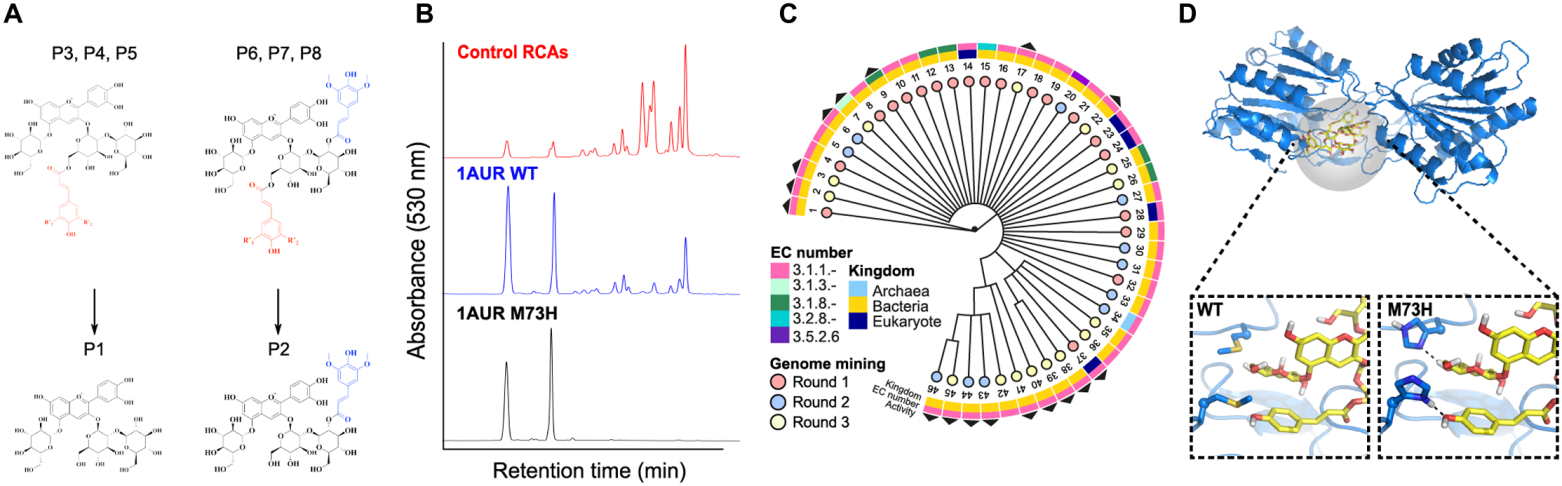
Enzyme discovery and design for transforming RCAs into P2. (**A**) Structural similarities between RCAs facilitate enzymatic enhancements of P1 and P2. Selective hydrolysis cleaving the acyl group from Glc-1 (red highlight) transforms P3, P4, and P5 into P1, and P6, P7, and P8 into P2. Selectivity toward the acyl group on Glc-1, but not the acyl group on Glc-2 (blue highlight), is critical for P2 production. (**B**) HPLC chromatograms of RCAs treated with different enzymes: RCAS without enzyme (red, top), RCAs after incubation with the native 1AUR WT protein (blue, middle), and RCAs after incubation with mutant 1AUR M73H (black, bottom). See method S11.2 for reaction conditions. (**C**) Phylogenetic tree of enzymes tested for activity on RCAs. Black triangles indicate that enzyme had desired hydrolytic activity. Additional metadata are provided in the tree and corresponding legend. See method S11.1 for methodology. (**D**) (Top) Cartoon depiction of 1AUR WT with P6 in the active site (yellow with gray highlight), with the protein backbone shown in blue. The predicted molecular interactions of the native amino acid (bottom left) and designed mutation (bottom right) with P6 are illustrated. Dashed lines from the introduced histidine depict the predicted new hydrogen bonds between the enzyme and anthocyanin, which we hypothesize contributes to the enhanced activity.

While the Al^3+^(P2^−^)_3_ complex has the unique chemical conformation that provides the spectral properties desired for replacing FD&C Blue No. 1, P2 represents <5% of the total anthocyanin content naturally occurring in red cabbage. Hence, an ambitious challenge was obtaining sufficient quantities of P2 at high purity for the present research program and for potential use as a food coloring agent. To that end, the homology of structures in red cabbage was leveraged and a hydrolytic enzyme capable of a highly selective catalytic deacylation was found and further developed. The enzyme selectively removes any acyl group bound to Glc-1 of the sophorose moiety while leaving the sinapoyl group on Glc-2 intact, thus converting P6, P7, and P8 into P2 (Fig. 4, A and B). Initial database mining efforts comprised screening a broad range of genes encoding hydrolytic enzymes, including lactonases, esterases, lactamases, and hydrolases, curated from the BRENDA database across 26 enzyme classifications (ECs) (Fig. 4C and tables S11.1 and S11.2) (*32*). Over three rounds of genomic mining, a total of 46 genes were tested, of which 17 were active on P6 to P8, converting them to P2 (method S11.2). The median sequence identity of the total set of tested enzymes is 7.0%, and the median sequence identity of active enzymes is 13.0% (Fig. 4C), highlighting the plasticity of functionality within the esterase family through the diversity of sequences capable of catalyzing this reaction.

The most active of these enzymes was 1AUR, which in its native form hydrolyzes longer fatty acid chains but has demonstrated broad specificity toward esters (fig. S11.1) (*33*). We subsequently redesigned the pocket of the 1AUR protein (*24*) using a combination of Rosetta Design and FoldIt to introduce new favorable interactions with P8 (the largest and most complex of the RCAs) (*34*, *35*). Across multiple rounds of design, the mutant with the best conversion of P6, P7, and P8 was M73H, which was able to drive the transformation of RCAs 3 to 8 to P1 and P2 to completion in a time frame and enzyme concentration viable for the gram-scale production of P2 needed for food-product prototyping (Fig. 4B, table S11.3, and figs. S11.2 and S11.3). On the basis of the modeling and design efforts, we hypothesize that the two additional hydrogen bonds that are developed by the histidine residue substituted at position 73, which are both exposed at the active cleft, strengthen the enzyme-substrate interaction (Fig. 4D).

### Color expression and application of P2

**Fig. 5:**
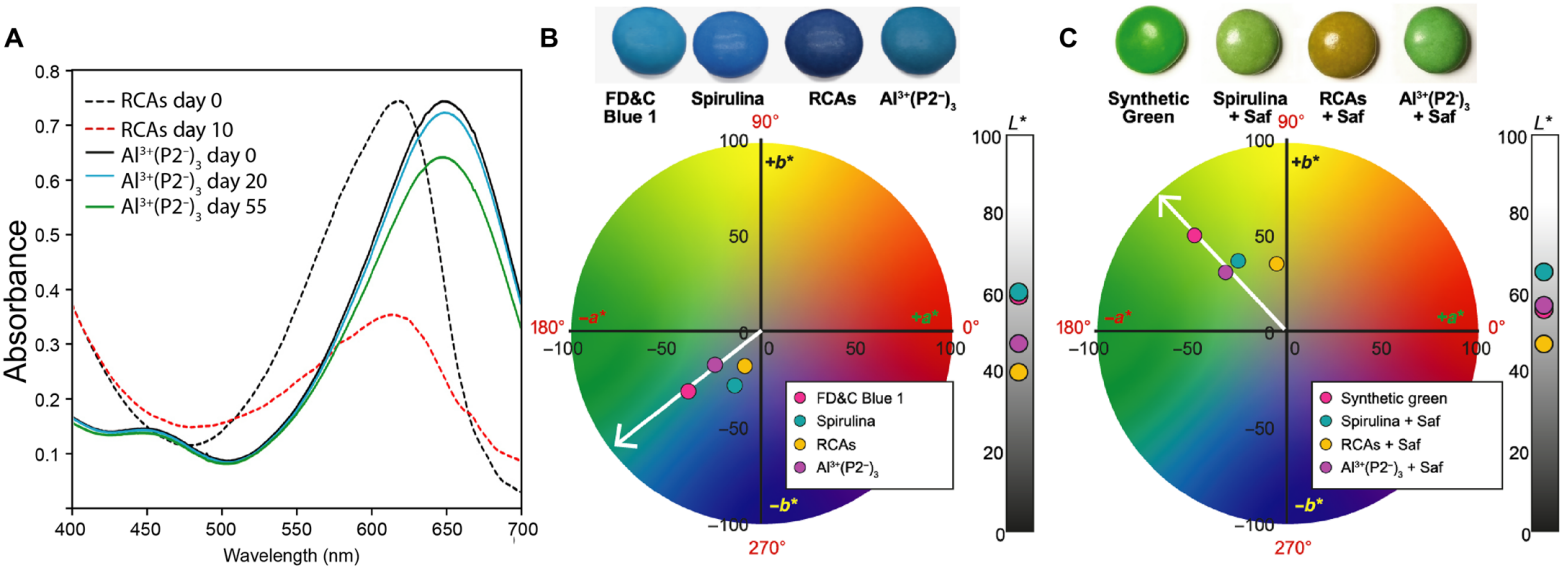
Product prototyping. (**A**) Visible absorption spectra of Al^3+^(P2^−^)_3_ at pH 7 (solid lines) and RCAs at pH 8 (dashed lines) in saturated sugar syrup monitored over time. Over the course of 10 days, the absorbance at λ_max_ of RCAs drops by 57%, indicating loss of cyanidin chromophore, whereas the Al^3+^(P2^−^)_3_ complex shows remarkable stability with only 14% loss of absorbance at λ_max_ over the course of 55 days. Experimental details are in section S13.3. (**B**) Sugar-coated lentils (top) using the four blue colorants were obtained under typical panning conditions. The colorants were FD&C Blue No. 1, spirulina, RCAs at pH 8, and Al^3+^(P2^−^)_3_ at pH 7. The colorimetry data are plotted in a*b* space and L* values in the sidebar. The Al^3+^(P2^−^)_3_ complex at pH 7 provides a very similar hue angle to FD&C Blue No. 1 (sections S1 and S13). (**C**) Sugar-coated lentils (top) where the colorant blends used were synthetic green, spirulina, red cabbage at pH 8, and Al^3+^(P2^−^)_3_ at pH 7, with the latter three mixed with safflower (Saf) as the yellow component. Al^3+^(P2^−^)_3_ at pH 7 with safflower provides a very similar hue angle to synthetic green (fig. S13.4 and table S13.4).

Following chromatographic purification of the enzymatic transformation and complex formation with Al^3+^ (methods S11.3 to S11.4 and section S12), gram quantities of the Al^3+^(P2^−^)_3_ complex were obtained, enabling detailed investigations of the color expression and stability of the novel colorant. In any food product application, color stability is critical. While many naturally sourced colorants, including anthocyanins, have limited stability over time, the Al^3+^(P2^−^)_3_ complex at pH 7 showed remarkable stability in sugar syrup over 55 days with only a 14% loss of color (Fig. 5A) (*19*). Its performance as a colorant was also demonstrated in application to create blue and green colors (hue angle match to a Christmas green shade; fig. S2.1) in several food and confectionery products (Fig. 5, B and C, figs. S13.1 to S13.4, and table S13.1 to S13.4). The stability of this novel colorant in these product applications is excellent as well, with no notable color decay over a 30-day period when stored at ambient conditions (fig. S13.5 and table S13.5). Storage under acidic conditions further enhances the stability of the colorant by reducing fraction of hydrolyzed species, although neutral pH must be able to be restored for integration into product (figs. S3.1 and S9.1) (*19*). While these initial studies provide a clear starting point for the development of a natural FD&C Blue No. 1, future efforts will be critical to evaluate both stability and color in a wide variety of applications to define usage limitations (discussion S12.1) and appropriate colorant and food safety precautions (*36*–*38*).

In summary, combining modern techniques from analytical chemistry, food science, biochemistry, synthetic biology, color science, and computational chemistry, we found, characterized, and defined a path to production for a naturally sourced cyan blue colorant whose color properties are nearly identical to those of the industry standard brilliant blue FCF (FD&C Blue No. 1). This colorant is also capable of producing superior green colors compared to many existing natural blue colorants. This discovery requiring a global collaboration provides a solution to a long-standing food color need, potentially fulfilling the growing consumer demands for utilization of more natural ingredients in food while keeping a vibrant color palette.

## MATERIALS AND METHODS

Detailed methods for the experiments conducted in this study can be found in the Supplementary Materials.

### Violet contribution calculation

The violet contribution of a blue colorant was defined as the area under the visible absorption curve (AUC). The area was calculated using the left Riemann sum for integration, where the height is the absorbance and the width is the spectral resolution (for more specific details, see method S1.1).

### Ultraviolet-visible spectrometry

Solutions were poured into relevant cuvette of 10-, 1-, and 0.1-mm cell length, and then ultraviolet (UV)–visible absorption spectra (200 to 800 nm) were recorded on a JASCO V-560 spectrophotometer. The solutions were kept at room temperature and protected from light (method S6.1).

### Measurement of CD

CD was measured from 200 to 800 nm with a JASCO J-720 spectrometer using the same solution for the measurement of stability described in section S6.1. To increase signal/noise ratio, the scan was repeated four times, and the data were averaged (method S7.1) (*21*).

### ESI-TOF-MS of aluminum complexes

ESI-MS spectra were recorded with a Bruker COMPACT instrument and analyzed with the application purchased from Bruker Daltonics (method S7.1).

### NMR measurement of aluminum complex of P2

NMR spectra were obtained with Bruker Daltonics AVANCE III HD 600 with a TCI cryoprobe and BBO cryoprobe (^1^H: 600 MHz and ^13^C: 150 MHz) in a 5–mm–inside diameter tube at variable temperatures in D_2_O. Chemical shifts were recorded as parts per million (ppm) using the proton resonance in the semi-heavy water (HDO) as a standard (4.67 ppm). Various 1D and 2D measurements were carried out (method S7.3).

### Geometric optimization of putative P2 and P5 3:1 complexes with aluminum

Optimizations of either P2 or P5 molecules in a 3:1 complex with aluminum were performed, starting from relevant structures of monomers obtained from extensive MD simulations and analysis, as detailed in section S10.1. These were arranged with a C3 symmetry around Al^3+^ and deprotonated at 3′ and 4′, and with Al^3+^ coordinated to the oxygens at 3′ and 4′. The two chiral assemblies of the ligands, corresponding to left and right handed (labeled Λ and Δ, respectively), were tried. Relaxations were then performed in two steps: (i) A preoptimization was run in vacuum with CP2K (*39*), with DZVP-MOLOPT-SR-GTH basis set and GTH-BLYP pseudopotential (*40*–*42*) corrected with the D3(0) Grimme dispersion (*43*). (ii) Final optimizations were run in Quantum Espresso (*44*, *45*), using ultrasoft pseudopotentials and the Perdew-Burke-Ernzerhof (PBE) exchange-correlation functional (*46*) with implicit solvent (Environ module) (*47*) in periodic cubic box of 80 bohr. The following parameters were adopted for the wavefunction convergence: kinetic-energy cutoff of 35 rydberg (Ry) and charge density cutoff of 320 Ry. The Makov-Payne energy correction was used to remove finite-box effects (method S8.1).

### MD simulations of P2 and P5

Classical MD simulations of P5 and P2 were run using GROMACS 4.5.5 (*48*) upon adequate equilibration with all bonds to hydrogen atoms constrained using LINCS (*49*). General AMBER force field (GAFF) parameters were assigned using the antechamber module of AmberTools13 (*50*, *51*) with RESP charges at the HF/6-31G* level, calculated on DFT-B3LYP optimized geometries, 50-/200-Ry basis set. For each species studied, 10 replicas were simulated using Hamiltonian replica-exchange MD (HREMD) (*52*) as implemented in the PLUMED plugin (*53*) (method S10.1).

### Sequence alignment and construction of the phylogenetic tree of esterases of interest

Sequences tested in this study (table S11.1) were aligned using Geneious 2017.10.1.3 using multiple sequence comparison by log-expectation (MUSCLE) alignment (method S11.1.).

The phylogenetic tree in Fig. 4C was constructed using the Geneious Consensus Tree Builder (method S11.1) and visualized using GraPhlAn (https://github.com/siegel-lab-ucd/blue-pigment-publication.git) (*54*).

### Protein purification and activity screening of esterases

An *Escherichia coli* codon-optimized gene encoding each protein was purchased from Twist Biosciences and transferred into pET29b^+^ to encode a C-terminal hexahistidine tag. Mutant plasmids were produced by Kunkel mutagenesis (*55*). Plasmids were incorporated into *E. coli* BL21(DE3) via electroporation. Cultures were grown in Terrific Broth at 37°C, induced with 1 mM isopropyl-β-d-thiogalactopyranoside, and allowed to express at 18°C for 24 hours, after which cells were lysed, clarified, and protein-purified using gravity columns with immobilized metal affinity chromatography, the details of which are provided in method S11.2. Proteins were screened for activity by combining 10 μl of RCE (100 mg/ml) with 90 μl of 50 mM Hepes buffer (pH 7.5) and allowed to proceed at room temperature for 24 hours. Reactions were quenched with 70% methanol and brought to pH 3 with 1 μl of HCl. Reactions were centrifuged at 4700 rpm for 3 min to remove insoluble protein. Analysis was done using the high-performance liquid chromatography (HPLC) method described in method S11.2.

### Gram-scale protein production

The M73H point mutant was created from the 1AUR (sequence ID no. 46) wild-type (WT) plasmid via Kunkel mutagenesis (*55*) and then transformed into chemically competent *E. coli* BLR (DE3) cells (details provided in method S11.3). The supernatant lysate containing active enzyme was collected and used for the reaction. The scaled-up generation of P2 via the enzymatic reaction is described in method S11.4.

### P2 purification from enzyme-treated red cabbage

The material was purified using several techniques to remove impurities: enzyme precipitation, solid-phase extraction, and preparatory HPLC (more detailed description is found in method S12.1).

### Anthocyanin-metal complex [Al^3+^(P2^−^)_3_] formation details

One-third equivalents of AlK(SO_4_)_2_ stock solution were added to the aqueous P2 solution, adjusted to 7.0. The P2-Al complex solution was transferred to a polypropylene container, immersed in liquid nitrogen until fully frozen, and then placed into the lyophilizer with vacuum set to 0.03 mbar and the condenser to −80°C. More detailed description is provided in method S12.2.

## SUPPLEMENTARY MATERIALS

Supplementary material for this article is available at http://advances.sciencemag.org/cgi/content/full/7/15/eabe7871/DC1

## References

[1] F. M. Clydesdale, Color as a factor in food choice. Crit. Rev. Food Sci. Nutr. 33, 83–101 (2009). 10.1080/10408399309527614 8424857

[2] D. Asioli, J. Aschemann-Witzel, V. Caputo, R. Vecchio, A. Annunziata, T. Næs, P. Varela, Making sense of the “clean label” trends: A review of consumer food choice behavior and discussion of industry implications. Food Res. Int. 99, 58–71 (2017). 28784520 10.1016/j.foodres.2017.07.022

[3] K. Kupferschmidt, In search of blue. Science 364, 424–429 (2019). 31048474 10.1126/science.364.6439.424

[4] B. C. Freitas-Dörr, C. O. Machado, A. C. Pinheiro, A. B. Fernandes, F. A. Dörr, E. Pinto, M. Lopes-Ferreira, M. Abdellah, J. Sá, L. C. Russo, F. L. Forti, L. C. P. Gonçalves, E. L. Bastos, A metal-free blue chromophore derived from plant pigments. Sci. Adv. 6, eaaz0421 (2020). 32284978 10.1126/sciadv.aaz0421 PMC7124932

[5] A. E. Smith, H. Mizoguchi, K. Delaney, N. A. Spaldin, A. W. Sleight, M. A. Subramanian, Mn^3+^ in trigonal bipyramidal coordination: A new blue chromophore. J. Am. Chem. Soc. 131, 17084–17086 (2009). 19899792 10.1021/ja9080666

[6] M. Buchweitz, Natural solutions for blue colors in food, in *Handbook on Natural Pigments in Food and Beverages: Industrial Applications for Improving Food Color*, R. Carle, R. M. Schweiggert, Eds. (Elsevier, 2016), chapter 17, pp. 355–384.

[7] P. Nabais, J. Oliveira, F. Pina, N. Teixeira, V. de Freitas, N. F. Brás, A. Clemente, M. Rangel, A. M. S. Silva, M. J. Melo, A 1000-year-old mystery solved: Unlocking the molecular structure for the medieval blue from *Chrozophora tinctoria*, also known as folium. Sci. Adv. 6, eaaz7772 (2020). 32426456 10.1126/sciadv.aaz7772 PMC7164948

[8] A. G. Newsome, C. A. Culver, R. B. van Breemen, Nature’s palette: The search for natural blue colorants. J. Agric. Food Chem. 62, 6498–6511 (2014). 24930897 10.1021/jf501419q

[9] L. Jespersen, L. D. Strømdahl, K. Olsen, L. H. Skibsted, Heat and light stability of three natural blue colorants for use in confectionary beverages. Eur. Food Res. Technol. 220, 261–266 (2004).

[10] A. Chaovanalikit, M. M. Thompson, R. E. Wrolstad, Characterization and quantification of anthocyanins and polyphenolics in blue honeysuckle (*Lonicera caerulea* L.). J. Agric. Food Chem. 52, 848–852 (2004). 14969540 10.1021/jf030509o

[11] G. T. Sigurdson, P. Tang, M. M. Giusti, Natural colorants: Food colorants from natural sources. Annu. Rev. Food Sci. Technol. 8, 261–280 (2017). 28125346 10.1146/annurev-food-030216-025923

[12] I. Viera, A. Pérez-Gálvez, M. Roca, Green natural colorants. Molecules 24, 154 (2019). 10.3390/molecules24010154 PMC6337735 30609768

[13] O. Dangles, J.-A. Fenger, The chemical reactivity of anthocyanins and its consequences in food science and nutrition. Molecules 23, 1970 (2018). 10.3390/molecules23081970 PMC6222895 30087225

[14] F. Pina, M. J. Melo, C. A. T. Laia, A. J. Parola, J. C. Lima, Chemistry and applications of flavylium compounds: A handful of colours. Chem. Soc. Rev. 42, 869–908 (2012). 10.1039/c1cs15126f 21842035

[15] P. Trouillas, J. C. Sancho-García, V. De Freitas, J. Gierschner, M. Otyepka, O. Dangles, Stabilizing and modulating color by copigmentation: Insights from theory and experiment. Chem. Rev. 116, 4937–4982 (2016). 26959943 10.1021/acs.chemrev.5b00507

[16] O. Dangles, N. Saito, R. Brouillard, Kinetic and thermodynamic control of flavylium hydration in the pelargonidin-cinnamic acid complexation. Origin of the extraordinary flower color diversity of Pharbitis nil. J. Am. Chem. Soc. 115, 3125–3132 (1993).

[17] K. Yoshida, M. Mori, T. Kondo, Blue flower color development by anthocyanins: From chemical structure to cell physiology. Nat. Prod. Rep. 26, 884–915 (2009). 19554240 10.1039/b800165k

[18] J.-A. Fenger, M. Moloney, R. J. Robbins, T. M. Collins, O. Dangles, The influence of acylation, metal binding and natural antioxidants on the thermal stability of red cabbage anthocyanins in neutral solution. Food Funct. 10, 6740–6751 (2019). 31576890 10.1039/c9fo01884k

[19] G. T. Sigurdson, R. J. Robbins, T. M. Collins, M. M. Giusti, Molar absorptivities (ε) and spectral and colorimetric characteristics of purple sweet potato anthocyanins. Food Chem. 271, 497–504 (2019). 30236708 10.1016/j.foodchem.2018.07.096

[20] N. Ahmadiani, R. J. Robbins, T. M. Collins, M. M. Giusti, Molar absorptivity (ε) and spectral characteristics of cyanidin-based anthocyanins from red cabbage. Food Chem. 197, 900–906 (2016). 26617032 10.1016/j.foodchem.2015.11.032

[21] M. Moloney, R. J. Robbins, T. M. Collins, T. Kondo, K. Yoshida, O. Dangles, Red cabbage anthocyanins: The influence of D-glucose acylation by hydroxycinnamic acids on their structural transformations in acidic to mildly alkaline conditions and on the resulting color. Dyes Pigments 158, 342–352 (2018).

[22] K. Ikeda, Structure of two acylated anthocyanins from red cabbage (*Brassica oleracea*). Chem. Express 2, 563–566 (1987).

[23] N. Saito, F. Tatsuzawa, E. Suenaga, K. Toki, K. Shinoda, A. Shigihara, T. Honda, Tetra-acylated cyanidin 3-sophoroside-5-glucosides from the flowers of *Iberis umbellata* L.(*Cruciferae*). Phytochemistry 69, 3139–3150 (2008). 18514755 10.1016/j.phytochem.2008.04.010

[24] R. Matera, S. Gabbanini, G. R. De Nicola, R. Iori, G. Petrillo, L. Valgimigli, Identification and analysis of isothiocyanates and new acylated anthocyanins in the juice of *Raphanus sativus* cv. Sango sprouts. Food Chem. 133, 563–572 (2012). 25683434 10.1016/j.foodchem.2012.01.050

[25] R. Matera, S. Gabbanini, S. Berretti, R. Amorati, G. R. De Nicola, R. Iori, L. Valgimigli, Acylated anthocyanins from sprouts of *Raphanus sativus* cv. Sango: Isolation, structure elucidation and antioxidant activity. Food Chem. 166, 397–406 (2015). 25053073 10.1016/j.foodchem.2014.06.056

[26] T. Ito, K.-i. Oyama, K. Yoshida, Direct observation of hydrangea blue-complex composed of 3-*O*-glucosyldelphinidin, Al^3+^ and 5-*O*-acylquinic acid by ESI-mass spectrometry. Molecules 23, 1424 (2018). 10.3390/molecules23061424 PMC6100629 29895788

[27] G. T. Sigurdson, R. J. Robbins, T. M. Collins, M. M. Giusti, Spectral and colorimetric characteristics of metal chelates of acylated cyanidin derivatives. Food Chem. 221, 1088–1095 (2017). 27979063 10.1016/j.foodchem.2016.11.052

[28] K. Takeda, Blue metal complex pigments involved in blue flower color. Proc. Jpn. Acad. Ser. B Phys. Biol. Sci. 82, 142–154 (2006). 10.2183/pjab.82.142 PMC4323046 25792777

[29] T. Kondo, M. Ueda, H. Tamura, K. Yoshida, M. Isobe, T. Goto, , Composition of protocyanin, a self-assembled supramolecular pigment from the blue cornflower, *Centaurea cyanus*. Angew. Chem. Int. 33, 978–979 (1994).

[30] M. Rusishvili, L. Grisanti, S. Laporte, M. Micciarelli, M. Rosa, R. J. Robbins, T. Collins, A. Magistrato, S. Baroni, Unraveling the molecular mechanisms of color expression in anthocyanins. Phys. Chem. Chem. Phys. 21, 8757–8766 (2019). 30968901 10.1039/c9cp00747d

[31] O. B. Malcıoğlu, A. Calzolari, R. Gebauer, D. Varsano, S. Baroni, Dielectric and thermal effects on the optical properties of natural dyes: A case study on solvated cyanin. J. Am. Chem. Soc. 133, 15425–15433 (2011). 21905678 10.1021/ja201733v

[32] L. Jeske, S. Placzek, I. Schomburg, A. Chang, D. Schomburg, BRENDA in 2019: A European ELIXIR core data resource. Nucleic Acids Res. 47, D542–D549 (2018). 10.1093/nar/gky1048 PMC6323942 30395242

[33] K. K. Kim, H. K. Song, D. H. Shin, K. Y. Hwang, S. Choe, O. J. Yoo, S. W. Suh, Crystal structure of carboxylesterase from *Pseudomonas fluorescens,* an α/β hydrolase with broad substrate specificity. Structure 5, 1571–1584 (1997). 9438866 10.1016/s0969-2126(97)00306-7

[34] R. Kleffner, J. Flatten, A. Leaver-Fay, D. Baker, J. B. Siegel, F. Khatib, S. Cooper, Foldit Standalone: A video game-derived protein structure manipulation interface using Rosetta. Bioinformatics 33, 2765–2767 (2017). 28481970 10.1093/bioinformatics/btx283 PMC5860063

[35] J. K. Leman, B. D. Weitzner, S. M. Lewis, J. Adolf-Bryfogle, N. Alam, R. F. Alford, M. Aprahamian, D. Baker, K. A. Barlow, P. Barth, B. Basanta, B. J. Bender, K. Blacklock, J. Bonet, S. E. Boyken, P. Bradley, C. Bystroff, P. Conway, S. Cooper, B. E. Correia, B. Coventry, R. Das, R. M. De Jong, F. D. Maio, L. Dsilva, R. Dunbrack, A. S. Ford, B. Frenz, D. Y. Fu, C. Geniesse, L. Goldschmidt, R. Gowthaman, J. J. Gray, D. Gront, S. Guffy, S. Horowitz, P.-S. Huang, T. Huber, T. M. Jacobs, J. R. Jeliazkov, D. K. Johnson, K. Kappel, J. Karanicolas, H. Khakzad, K. R. Khar, S. D. Khare, F. Khatib, A. Khramushin, I. C. King, R. Kleffner, B. Koepnick, T. Kortemme, G. Kuenze, B. Kuhlman, D. Kuroda, J. W. Labonte, J. K. Lai, G. Lapidoth, A. Leaver-Fay, S. Lindert, T. Linsky, N. London, J. H. Lubin, S. Lyskov, J. Maguire, L. Malmström, E. Marcos, O. Marcu, N. A. Marze, J. Meiler, R. Moretti, V. K. Mulligan, S. Nerli, C. Norn, S. Ó’Conchúir, N. Ollikainen, S. Ovchinnikov, M. S. Pacella, X. Pan, H. Park, R. E. Pavlovicz, M. Pethe, B. G. Pierce, K. B. Pilla, B. Raveh, P. D. Renfrew, S. S. Roy Burman, A. Rubenstein, M. F. Sauer, A. Scheck, W. Schief, O. Schueler-Furman, Y. Sedan, A. M. Sevy, N. G. Sgourakis, L. Shi, J. B. Siegel, D.-A. Silva, S. Smith, Y. Song, A. Stein, M. Szegedy, F. D. Teets, S. B. Thyme, R. Y.-R. Wang, A. Watkins, L. Zimmerman, R. Bonneau, Macromolecular modeling and design in Rosetta: Recent methods and frameworks. Nat. Methods 17, 665–680 (2020). 32483333 10.1038/s41592-020-0848-2 PMC7603796

[36] R. E. Wrolstad, C. A. Culver, Alternatives to those artificial FD&C food colorants. Annu. Rev. Food Sci. Technol. 3, 59–77 (2012). 22385164 10.1146/annurev-food-022811-101118

[37] U.S. Food and Drug Administration (FDA), *Summary of Color Additives for Use in the United States in Foods, Drugs, Cosmetics, and Medical Devices* (EPA, 2015); https://www.fda.gov/industry/color-additive-inventories/summary-color-additives-use-united-states-foods-drugs-cosmetics-and-medical-devices.

[38] M. W. Pariza, E. M. Foster, Determining the safety of enzymes used in food processing. J. Food Prot. 46, 453–468 (1983). 30913657 10.4315/0362-028X-46.5.453

[39] J. Hutter, M. Iannuzzi, F. Schiffmann, J. V. Vondele, CP2K: Atomistic simulations of con-densed matter systems. WIREs Comput. Mol. Sci. 4, 15–25 (2014).

[40] S. Goedecker, M. Teter, J. Hutter, Eparable dual-space gaussian pseudopotentials. Phys.Rev. B 54, 1703–1710 (1996). 10.1103/physrevb.54.1703 9986014

[41] A. D. Becke, Density-functional exchange-energy approximation with correct asymptotic behavior. Phys. Rev. A 38, 3098–3100 (1988). 10.1103/physreva.38.3098 9900728

[42] C. Lee, W. Yang, R. G. Parr, Development of the Colle-Salvetti correlation-energy formula into a functional of the electron density. Phys. Rev. B 37, 785 (1988). 10.1103/physrevb.37.785 9944570

[43] S. Grimme, J. Antony, S. Ehrlich, H. Krieg, A consistent and accurate *ab initio* parametrization of density functional dispersion correction (DFT-D) for the 94 elements H-Pu. J. Chem. Phys. 143, 154104 (2010). 10.1063/1.3382344 20423165

[44] P. Giannozzi, S. Baroni, N. Bonini, M. Calandra, R. Car, C. Cavazzoni, D. Ceresoli, G. L. Chiarotti, M. Cococcioni, I. Dabo, A. D. Corso, S. de Gironcoli, S. Fabris, G. Fratesi, R. Gebauer, U. Gerstmann, C. Gougoussis, A. Kokalj, M. Lazzeri, L. Martin-Samos, N. Marzari, F. Mauri, R. Mazzarello, S. Paolini, A. Pasquarello, L. Paulatto, C. Sbraccia, S. Scandolo, G. Sclauzero, A. P. Seitsonen, A. Smogunov, P. Umari, R. M. Wentzcovitch, QUANTUM ESPRESSO: A modular and open-source software project for quantum simulations of materials. J. Condens. Matter Phys. 21, 395502 (2009). 10.1088/0953-8984/21/39/395502 21832390

[45] P. Giannozzi, O. Andreussi, T. Brumme, O. Bunau, M. B. Nardelli, M. Calandra, R. Car, C. Cavazzoni, D. Ceresoli, M. Cococcioni, N. Colonna, I. Carnimeo, A. D. Corso, S. de Gironcoli, P. Delugas, R. A. Di Stasio Jr., A. Ferretti, A. Floris, G. Fratesi, G. Fugallo, R. Gebauer, U. Gerstmann, F. Giustino, T. Gorni, J. Jia, M. Kawamura, H.-Y. Ko, A. Kokalj, E. Küçükbenli, M. Lazzeri, M. Marsili, N. Marzari, F. Mauri, N. L. Nguyen, H.-V. Nguyen, A. Otero-de-la-Roza, L. Paulatto, S. Poncé, D. Rocca, R. Sabatini, B. Santra, M. Schlipf, A. P. Seitsonen, A. Smogunov, I. Timrov, T. Thonhauser, P. Umari, N. Vast, X. Wu, S. Baroni, Advanced capabilities for materials modelling with Quantum ESPRESSO. J. Phys. Condens. Matter 29, 465901 (2017). 29064822 10.1088/1361-648X/aa8f79

[46] J. P. Perdew, K. Burke, M. Ernzerhof, Generalized gradient approximation made simple. Phys. Rev. Lett. 77, 3865–3868 (1996). 10062328 10.1103/PhysRevLett.77.3865

[47] O. Andreussi, I. Dabo, N. Marzari, Revised self-consistent continuum solvation in electronic-structure calculations. J. Chem. Phys. 136, 064102 (2012). 22360164 10.1063/1.3676407

[48] S. Pronk, S. Páll, R. Schulz, P. Larsson, P. Bjelkmar, R. Apostolov, M. R. Shirts, J. C. Smith, P. M. Kasson, D. van der Spoel, B. Hess, E. Lindahl, GROMACS 4.5: A high-throughput and highly parallel open source molecular simulation toolkit. Bioinformatics 29, 845–854 (2013). 23407358 10.1093/bioinformatics/btt055 PMC3605599

[49] B. Hess, H. Bekker, H. J. C. Berendsen, J. G. E. M. Fraaije, LINCS: A linear constraint solver for molecular simulations. J. Comput. Chem. 18, 1463–1472 (1997).

[50] J. Wang, W. Wang, P. A. Kollman, D. A. Case, Automatic atom type and bond type perception in molecular mechanical calculations. J. Mol. Graph. Model. 25, 247–260 (2006). 16458552 10.1016/j.jmgm.2005.12.005

[51] J. Wang, R. M. Wolf, J. W. Caldwell, P. A. Kollman, D. A. Case, Development and testing of a general AMBER force field. J. Comput. Chem. 25, 1157–1174 (2004). 15116359 10.1002/jcc.20035

[52] G. Bussi, Hamiltonian replica exchange in GROMACS: A flexible implementation. Mol. Phys. 112, 379–384 (2014).

[53] G. A. Tribello, M. Bonomi, D. Branduardi, C. Camilloni, G. Bussi, PLUMED 2: New feathers for an old bird. Comput. Phys. Commun. 185, 604–613 (2014).

[54] F. Ascinar, G. Weingart, T. L. Tickle, C. Huttenhower, N. Segata, Compact graphical representation of phylogenetic data and metadata with GraPhlAn. PeerJ 3, e1029 (2015). 26157614 10.7717/peerj.1029 PMC4476132

[55] T. A. Kunkel, Rapid and efficient site-specific mutagenesis without phenotypic selection. Proc. Natl. Acad. Sci. U.S.A. 82, 488–492 (1985). 3881765 10.1073/pnas.82.2.488 PMC397064

[56] K. Yoshida, T. Kondo, T. Goto, Intramolecular stacking conformation of gentiodelphin, a diacylated anthocyanin from *Gentiana makinoi*. Tetrahedron 42, 4313–4326 (1992).

[57] K. Yoshida, K.-i. Oyama, T. Kondo, Structure of polyacylated anthocyanins and their UV protective effect. Rec. Adv. Polyphen. Res. 5, 171–192 (2017).

[58] K. Yoshida, T. Kondo, T. Goto, Unusually stable monoacylated anthocyanin from purple yam *Dioscorea alata*. Tetrahedron Lett. 32, 5579–5580 (1991).

[59] J. K. Barton, A. Danishefsky, J. Goldberg, Tris (phenanthroline) ruthenium (II): Stereoselectivity in binding to DNA. JACS 106, 2172–2176 (1984).

[60] R. H. Byrd, P. Lu, J. Nocedal, C. Zhu, A limited memory algorithm for bound constrained optimization. SIAM J. Sci. Comput. 16, 1190–1208 (1995).

[61] J. VandeVondele, M. Krack, F. Mohamed, M. Parrinello, T. Chassaing, J. Hutter, Quickstep: Fast and accurate density functional calculations using a mixed Gaussian and plane waves approach. Comput. Phys. Commun. 167, 103–128 (2005).

[62] J. Ridley, M. Zerner, An intermediate neglect of differential overlap technique for spectroscopy: Pyrrole and the azines. Theor. Chim. Acta 32, 111–134 (1973).

[63] F. Neese, The ORCA program system. Wiley Interidscip. Rev. Comput. Mol. Sci. 2, 73–78 (2012).

[64] F. Neese, Software update: The ORCA program system, version 4.0. Wiley Interdiscip. Rev. Comput. Mol. Sci. 8, e1327 (2017).

[65] G. Bussi, D. Donadio, M. Parrinello, Canonical sampling through velocity rescaling. J. Chem. Phys. 126, 014101 (2007). 17212484 10.1063/1.2408420

[66] A. Rodriguez, A. Laio, Clustering by fast search and find of density peaks. Science 344, 1492–1496 (2014). 24970081 10.1126/science.1242072

[67] T. Stahl, H. Taschan, H. Brunn, Aluminum content of selected foods and food products. Environ. Sci. Eur. 23, 37 (2011).

[68] S. M. Sayied, R. A. Yokel, Aluminum content of some foods and food products in the USA, with aluminum food additives. Food Addit. Contam. 22, 234–244 (2005). 16019791 10.1080/02652030500073584

